# Supplementary material for: Analysis of Novel Immunological Biomarkers Related to Rheumatoid Arthritis Disease Severity
Source: Int J Mol Sci. 2023 Aug 2;24(15):12351. doi: 10.3390/ijms241512351 (PMC10418816; doi:10.3390/ijms241512351)
Supplement: Supplementary file 1 [file ijms-24-12351-s001.zip › Figures S1 and S2.pdf]

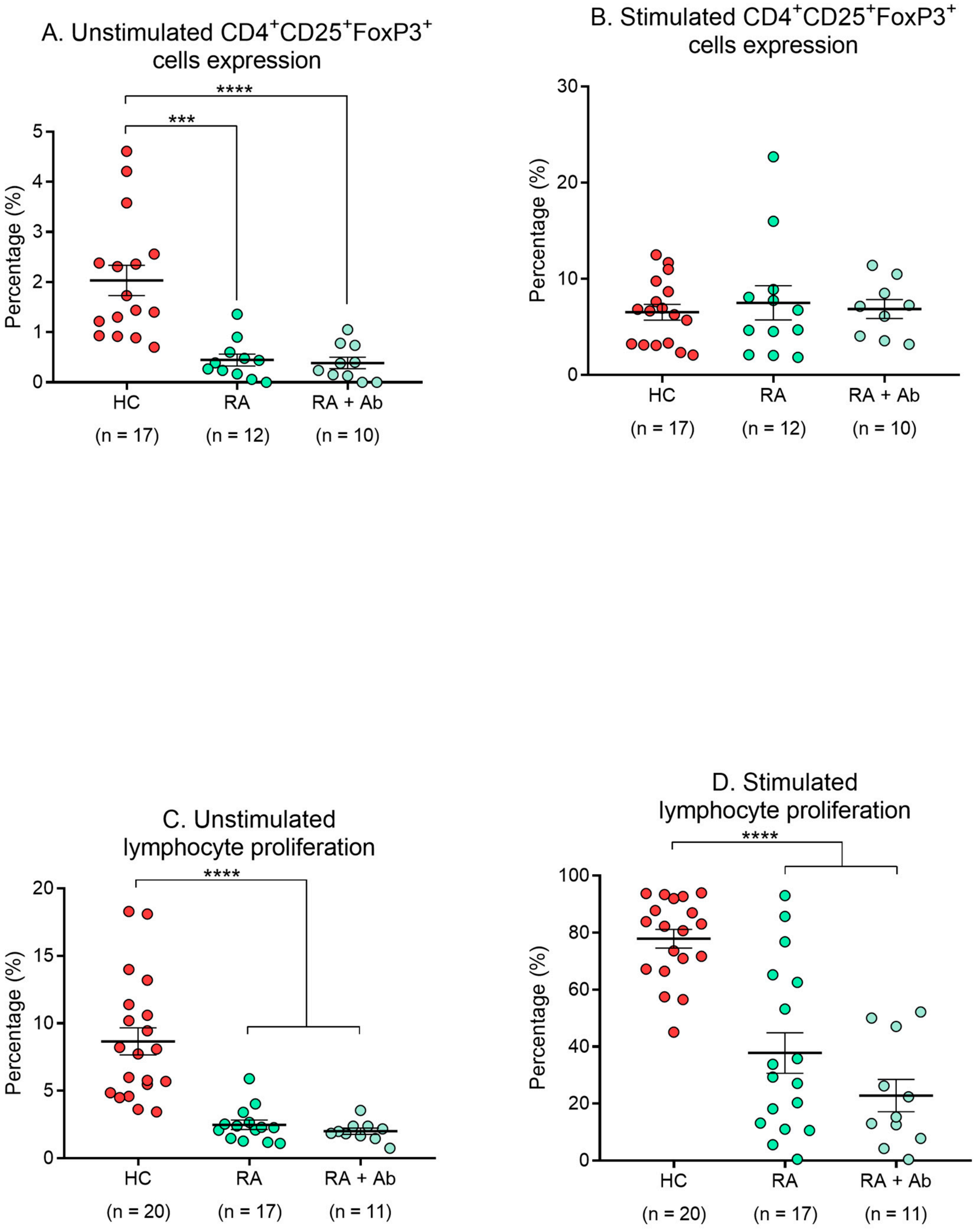

Figure S1: Analysis of T regulatory cell expression and lymphocyte proliferation. Percentage of unstimulated $CD4^+CD25^+FoxP3^+$ Treg cells (A) and stimulated $CD4^+CD25^+FoxP3^+$ Treg cells (B) in HCs, RA patients, and RA subjects given adalimumab ex vivo. Percentage of proliferating non-stimulated (C) and stimulated (D) lymphocytes was measured in HCs, RA patients, and RA subjects given adalimumab ex vivo. Bars show mean ± SEM. *p*-values were calculated using ANOVA (Fisher's LSD test) and the Kruskal–Wallis test (uncorrected Dunn's test). $^{***} = p < 0.001$; $^{****} = p < 0.0001$.

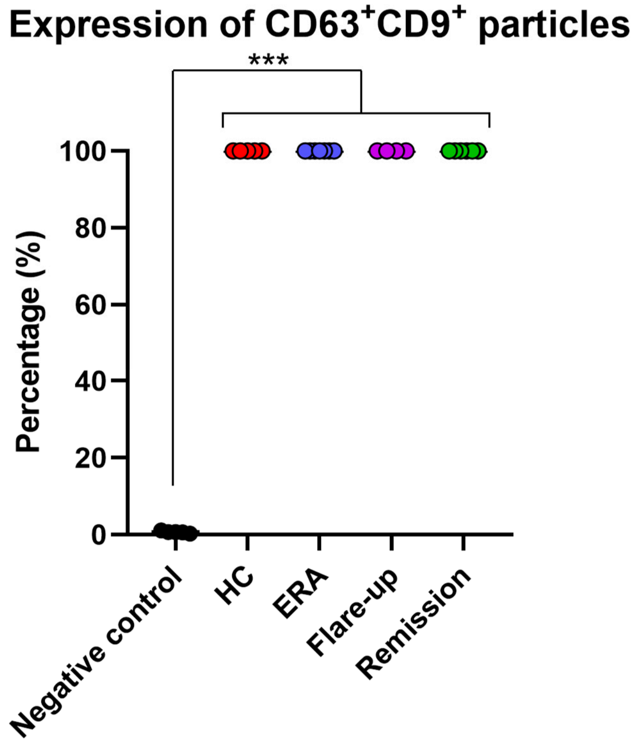

Figure S2: Analysis of CD63 and CD9 expression in exosomes. Percentage of CD63^{+}CD9^{+} expression in exosomes isolated from a negative control (PBS 1X), HCs, ERA, flare-up, and remission patients. *p*-values were calculated using the Kruskal–Wallis test (uncorrected Dunn's test). $^{***} = p < 0.001$.
