# Supplementary material for: Analysis of Novel Immunological Biomarkers Related to Rheumatoid Arthritis Disease Severity
Source: Int J Mol Sci. 2023 Aug 2;24(15):12351. doi: 10.3390/ijms241512351 (PMC10418816; doi:10.3390/ijms241512351)
Supplement: Supplementary file 1 [file ijms-24-12351-s001.zip › Table S1.pdf]

Table S1: Analysis of leukocyte populations. Percentage of total lymphocytes, T lymphocytes, CD3+CD4- lymphocytes, Th, NK cells, B lymphocytes, monocytes, and granulocytes and NK cells/T lymphocyte ratio in HCs, RA, ERA, flare-up, and remission patients. Results expressed as mean ± SEM. p-values were calculated using ANOVA (Fisher's LSD test) and the Kruskal–Wallis test (uncorrected Dunn's test). * Statistical differences.

Analysis of leukocyte populations (%)												
	Group					*p*-values						
	HC	RA	ERA	FU	REM	HC vs. RA	HC vs. ERA	HC vs. FU	HC vs. REM	ERA vs. FU	ERA vs. REM	FU vs. REM
n	21	19	7	5	7							
Total lymphocytes	34.4±1.6	23.02±2.01	23.36±4.2	20.32±4.5	24.6±2.1	<0.0001*	0.0038*	0.0014*	0.0093*	0.53	0.78	0.38
T lymphocytes	69.86±1.8	70.39±2.78	75±4.2	76.3±1.8	61.57±4.8	0.81	0.27	0.13	0.06	0.64	0.015*	0.0074*
$CD3^{+}CD4^{-}$ lymphocytes	24.48±1.7	27.86±2.67	27.94±4.1	36.56±5.9	21.56±3.1	0.28	0.38	0.0108*	0.46	0.11	0.19	0.0074*
Th lymphocytes	40.27±1.5	39.46±2.32	42.44±4.5	37.2±4.9	38.09±3.2	0.77	0.57	0.48	0.56	0.31	0.35	0.86
NK cells	15.7±1.8	12,99±1.96	11.26±2.6	8.46±2.2	17.96±3.9	0.17	0.2	0.0403*	0.82	0.43	0.22	0.0562
NK/T cells ratio	0.27±0.03	0.22±0.05	0.16±0.04	0.11±0.03	0.35±0.13	0.24	0.24	0.0422*	0.64	0.4	0.18	0.038*
B lymphocytes	7.88±0.7	8.19±0.69	8.243±1.5	8.1±0.9	8.2±1.2	0.76	0.79	0.89	0.82	0.94	0.98	0.95
Monocytes	6.13±0.6	6.31±0.53	5.843±0.7	6.92±1.1	6.33±1.1	0.81	0.58	0.65	0.77	0.42	0.83	0.54
Granulocytes	46.7±2.1	55.77±3.04	55.94±5.6	61.72±6.7	51.36±3.9	0.017*	0.072	0.0121*	0.36	0.39	0.46	0.13
