# Supplementary material for: Analysis of Novel Immunological Biomarkers Related to Rheumatoid Arthritis Disease Severity
Source: Int J Mol Sci. 2023 Aug 2;24(15):12351. doi: 10.3390/ijms241512351 (PMC10418816; doi:10.3390/ijms241512351)
Supplement: Supplementary file 1 [file ijms-24-12351-s001.zip › Table S2.pdf]

Table S2: Expression of leukocyte antigens. Percentage of expression of EGFR, EPCR, CD16, CD32, CD64, CD45RA, CD45RO, CD62L, and CD11a in lymphocyte subpopulations, monocytes, and granulocytes from HCs, ERA, flare-up, and remission patients. Results expressed as mean ± SEM.

Expression of leukocyte antigens (%)					
	Group				
	HC	RA	ERA	Flare-up	Remission
n	21	19	7	5	7
EGFR^{+} total lymphocytes	31.20±5.33	6.44±1.91	2.38±0.58	10.98±6.34	7.27±2.18
EPCR^{+} total lymphocytes	23.07±5.715	18.44±5.36	4.99±1.54	35.62±14.98	19.63±7.67
CD16^{+} total lymphocytes	40.26±4.97	44.43±4.93	29.56±4.86	66.96±11.47	43.2±3.77
CD32^{+} total lymphocytes	17.9±2.15	35.27±4.11	23.21±8.48	44.96±6.44	40.4±2.45
CD64^{+} total lymphocytes	10.27±1.62	9.24±2.45	5.97±2.94	11.7±4.73	10.76±5.18
CD45RA^{+} total lymphocytes	76.88±1.74	45.77±5.16	29.9±2.83	74.22±9.77	41.33±4.63
CD45RO^{+} total lymphocytes	42.42±2.24	68.07±4.43	74.7±3.96	48.02±12.04	75.77±3.19
CD62L^{+} total lymphocytes	76.96±2.82	58.16±3.94	63.14±6.32	43.32±7.72	63.77±4.74
CD11a^{+} total lymphocytes	84.06±2.65	46.89±5.79	33.77±8.41	69.06±9.73	44.19±8.31
EGFR^{+} T lymphocytes	39.6±6.72	5.73±1.94	2.24±0.60	10.68±6.52	5.69±2.26
EPCR^{+} T lymphocytes	30.70±8.66	19.26±6.1	3.93±1.42	39.66±16.42	20.01±8.97
CD16^{+} T lymphocytes	31.43±7.39	36.47±6.07	21.8±3.415	66.64±13.93	29.59±6.64
CD32^{+} T lymphocytes	3.84±1.06	32.58±4.74	10.89±3.28	44.4±8.47	45.84±3.54
CD64^{+} T lymphocytes	13.47±2.29	6.45±2.11	2.5±0.65	8.48±4.24	8.94±4.81
CD45RA^{+} T lymphocytes	76.7±1.98	54.79±4.81	39.39±3.58	80.2±9.1	52.06±4.29
CD45RO^{+} T lymphocytes	54.89±2.92	70.63±4.09	76.4±2.99	53.18±11.83	77.33±3.35
CD62L^{+} T lymphocytes	82.96±3.26	62.84±4.26	66.4±6.8	44.06±7.54	72.69±3.81
CD11a^{+} T lymphocytes	98.81±0.69	49.55±6.59	35.8±9.711	75.7±10.62	44.61±9.42
EGFR^{+} Th lymphocytes	41.07±6.97	5.62±1.9	2.19±0.6	10.46±6.22	5.6±2.43
EPCR^{+} Th lymphocytes	30.82±8.75	19.59±6.07	4.03±1.470	39.44±16.47	20.97±8.99
CD45RA^{+} Th lymphocytes	65.92±2.88	60.85±4.25	51.13±5.86	74.6±10.53	60.76±4.67
CD45RO^{+} Th lymphocytes	63.96±3.44	67.26±3.4	65.6±5.15	64.26±10.13	71.07±3.84
CD62L^{+} Th lymphocytes	85.8±3.17	69.04±5.5	80.06±4.22	37.32±11.26	80.67±2.28
CD11a^{+} Th lymphocytes	98.68±0.81	41.01±7.52	23.16±7.37	74.2±14.16	35.14±10.98
EGFR^{+} CD3^{+}CD4^{-} lymphocytes	37.68±6.48	5.59±2	2.19±0.61	10.68±6.97	5.36±2.05
EPCR^{+} CD3^{+}CD4^{-} lymphocytes	29.54±8.58	17.42±6.09	3.61±1.3	36.42±17.5	17.66±8.95
CD45RA^{+} CD3^{+}CD4^{-} lymphocytes	90.6±1.18	45.91±6.55	27.24±4.72	84.1±10.79	37.29±5.27
CD45RO^{+} CD3^{+}CD4^{-} lymphocytes	43.94±3.47	76.87±6.25	88.11±4.86	43.92±15.08	89.17±3.18
CD62L^{+} CD3^{+}CD4^{-} lymphocytes	78.32±4.18	53.13±4.21	47.07±8.13	54.58±5.77	58.14±7.22
CD11a^{+} CD3^{+}CD4^{-} lymphocytes	99.33±0.38	60.73±6.67	55.16±13.93	69.18±14.19	60.26±7.68
EGFR^{+} NK cells	22.34±5.14	7.43±1.99	3.27±1.11	13.54±6.37	7.23±2.05
EPCR^{+} NK cells	10.33±1.66	6.58±2.89	2.64±1.07	7.6±5.21	9.79±7.05
CD16^{+} NK cells	72.65±2.97	64.86±4.49	59.36±9.63	70.40±5.98	66.41±6.74
CD32^{+} NK cells	53.66±4.93	36.89±4.92	34.19±10.17	52.30±8.761	28.6±4.02
CD64^{+} NK cells	6.54±1.4	15.46±3.55	9.77±3.58	22.66±7.33	16.01±7.25
CD45RA^{+} NK cells	98.87±0.36	27.21±7.83	7.99±3.48	62.74±20.5	21.06±8.29
CD45RO^{+} NK cells	9.36±2.58	81.51±7.64	97.83±1.42	36.68±17.52	97.21±1.49
CD62L^{+} NK cells	74.42±3.97	39.65±5.07	38.01±6.8	56.7±13.83	29.11±4.32
CD11a^{+} NK cells	95.67±3.79	66.98±6.55	64.04±14.59	51.2±8.8	81.19±5.93
EGFR^{+} B lymphocytes	1.37±0.34	9.14±2.65	2.443±0.789	17.10±6.67	10.14±4.48
EPCR^{+} B lymphocytes	3.24±0.73	25.23±7.35	20.5±7.36	36.3±19.77	22.06±13.41
CD45RA^{+} B lymphocytes	99.75±0.095	28.04±8.9	4.9±2.16	81.24±18.49	13.19±3.83
CD45RO^{+} B lymphocytes	3.88±2.12	81.36±7.92	98.34±1.38	34.92±18.08	97.56±2.13
CD62L^{+} B lymphocytes	92.57±1.1	71.83±8.06	84.49±6.87	26.7±17.36	91.41±2.2
CD11a^{+} B lymphocytes	2.71±0.74	23.81±8.72	1.87±0.8	75.66±18.48	8.7±4.11
EGFR^{+} monocytes	53.78±7.77	22.72±6.6	21.04±13.24	27.18±14.37	21.2±8.81
EPCR^{+} monocytes	32.95±5.31	17.95±5.41	21.67±12.86	14.10±5.23	16.97±7.45
CD16^{+} monocytes	76.26±4.44	54.62±4.18	65.14±5.26	57.46±3.99	42.07±7.81
CD32^{+} monocytes	42.98±5.72	88.29±4.07	77.74±9.78	95.16±3.35	93.94±2.06

$CD64^+$ monocytes	83.93±2.42	73.02±4.99	67.83±9.44	87.68±4.39	67.73±8.34
$CD45RA^+$ monocytes	52.7±4	42.11±6.44	39.59±11.87	45.56±10.86	42.16±11.75
$CD45RO^+$ monocytes	56.88±4.78	47.11±4.8	43.24±6.36	43.78±15.61	53.34±4.54
$CD62L^+$ monocytes	97.07±0.51	81.25±4.28	89.09±2.21	69.06±15.1	82.13±2.74
$CD11a^+$ monocytes	97.62±0.47	61.51±6.43	52.56±11.66	74.64±6.39	61.09±12.13
$EGFR^+$ granulocytes	93.63±3.33	28.56±7.41	25.09±13.24	32.48±15.69	29.23±12.28
$EPCR^+$ granulocytes	47.33±7.14	17.17±6.08	19.29±13.17	13.5±5.97	17.7±10.42
$CD16^+$ granulocytes	99.03±0.34	96.72±1.15	98.31±0.36	97.28±0.77	94.73±3.04
$CD32^+$ granulocytes	92.15±4.18	79.46±6.01	74.47±11.46	77.2±14.56	86.07±7.06
$CD64^+$ granulocytes	28.94±5.31	50.23±7.13	38.79±12.66	57.62±15.5	56.4±10.22
$CD45RA^+$ granulocytes	23.42±2.81	20.86±5.78	20.43±11.94	21.42±9.01	20.89±9.52
$CD45RO^+$ granulocytes	64.58±4.67	49.01±7.58	41.63±12.26	50.14±18.96	55.57±11.41
$CD62L^+$ granulocytes	99.23±0.45	80.34±7.92	97.36±1.08	32.94±17.52	97.19±0.94
$CD11a^+$ granulocytes	97.65±0.53	32.04±8.7	7.58±2.354	78.7±17.75	23.19±9.38
