# Supplementary material for: Analysis of Novel Immunological Biomarkers Related to Rheumatoid Arthritis Disease Severity
Source: Int J Mol Sci. 2023 Aug 2;24(15):12351. doi: 10.3390/ijms241512351 (PMC10418816; doi:10.3390/ijms241512351)
Supplement: Supplementary file 1 [file ijms-24-12351-s001.zip › Table S3.pdf]

Table S3: Percentage of Treg cells and proliferating lymphocytes. Analysis of $CD4^+CD25^+FoxP3^+$ Treg cells (A) and lymphocyte proliferation (B) under unstimulated and stimulated conditions in HCs, RA, and RA+Ab. Results are expressed as mean ± SEM. p-values were calculated using ANOVA (Fisher's LSD test) and the Kruskal–Wallis test (uncorrected Dunn's test). * Statistical differences.

Percentage of Treg cells and proliferating lymphocytes						
	Group			*p*-values		
	HC	RA	RA+Ab	HC vs. RA	HC vs. RA+Ab	RA vs. RA+Ab
A. Analysis of $CD4^+CD25^+FoxP3^+$ Treg percentage						
n	17	12	9			
Unstimulated Treg cells	2.034±0.3	0.4464±0.12	0.387±0.11	0.0001*	<0.0001*	0.7416
Stimulated Treg cells	6.529±0.82	7.509±1.8	6.861±0.98	0.9617	0.6812	0.6707
B. Analysis of lymphocyte proliferation						
n	20	14	10			
Unstimulated lymphocytes	8.66±1	2.476±0.34	2±0.23	<0.0001*	<0.0001*	0.507
Stimulated lymphocytes	77.88±3.31	37.76±7.14	22.84±5.67	<0.0001*	<0.0001*	0.0855
