# Supplementary material for: Analysis of Novel Immunological Biomarkers Related to Rheumatoid Arthritis Disease Severity
Source: Int J Mol Sci. 2023 Aug 2;24(15):12351. doi: 10.3390/ijms241512351 (PMC10418816; doi:10.3390/ijms241512351)
Supplement: Supplementary file 1 [file ijms-24-12351-s001.zip › Table S4.pdf]

Table S4: Analysis of the expression of exosomal miRNAs in serum samples. Expression of miR-21, miR-146a, and miR-155 in HCs, flare-up, and remission patients. Results are expressed as mean ± SEM. p-values were calculated using 2-way ANOVA with the Tukey comparison test.

Analysis of the expression of exosomal miRNAs in serum samples						
	Group			*p*-values		
	ERA	Flare-up	Remission	ERA vs. flare-up	ERA vs. remission	Flare-up vs. remission
n	5	5	5			
miR-21	0.79±0.85	1.67±1.18	1.22±0.64	0.9988	0.9972	>0.9999
miR-146a	0.04	0.07	0.22	0.9988	0.9972	>0.9999
miR-155	0.17±0.91	0.34±0.88	0.71±0.52	0.9988	0.9972	>0.9999
